# Post‐exercise syncope: Wingate syncope test and visual‐cognitive function

**DOI:** 10.14814/phy2.12883

**Published:** 2016-08-22

**Authors:** Dylan C. Sieck, Matthew R. Ely, Steven A. Romero, Meredith J. Luttrell, Pedro M. Abdala, John R. Halliwill

**Affiliations:** ^1^ Department of Human PhysiologyUniversity of OregonEugeneOregon

**Keywords:** Anaerobic exercise, cerebrovascular circulation, hypotension, orthostatic, orthostatic intolerance, post‐exercise hypotension, syncope, tilt‐table test, vasovagal

## Abstract

Adequate cerebral perfusion is necessary to maintain consciousness in upright humans. Following maximal anaerobic exercise, cerebral perfusion can become compromised and result in syncope. It is unknown whether post‐exercise reductions in cerebral perfusion can lead to visual‐cognitive deficits prior to the onset of syncope, which would be of concern for emergency workers and warfighters, where critical decision making and intense physical activity are combined. Therefore, the purpose of this experiment was to determine if reductions in cerebral blood velocity, induced by maximal anaerobic exercise and head‐up tilt, result in visual‐cognitive deficits prior to the onset of syncope. Nineteen sedentary to recreationally active volunteers completed a symptom‐limited 60° head‐up tilt for 16 min before and up to 16 min after a 60 sec Wingate test. Blood velocity of the middle cerebral artery was measured using transcranial Doppler ultrasound and a visual decision‐reaction time test was assessed, with independent analysis of peripheral and central visual field responses. Cerebral blood velocity was 12.7 ± 4.0% lower (mean ± SE;* P *<* *0.05) after exercise compared to pre‐exercise. This was associated with a 63 ± 29% increase (*P *<* *0.05) in error rate for responses to cues provided to the peripheral visual field, without affecting central visual field error rates (*P *=* *0.46) or decision‐reaction times for either visual field. These data suggest that the reduction in cerebral blood velocity following maximal anaerobic exercise contributes to visual‐cognitive deficits in the peripheral visual field without an apparent affect to the central visual field.

## Introduction

Post‐exercise syncope is a transient loss of consciousness following an acute bout of exercise. The causes of post‐exercise syncope are multifactorial, but ultimately result in loss of consciousness due to reductions in cerebral perfusion (Halliwill et al. 2014). Before loss of consciousness occurs there is overt symptomatology of an impending faint, known as presyncope, that presents in most situations (Van Lieshout et al. 2003). It is estimated that 50–80% of healthy individuals will develop presyncopal signs and symptoms if subjected to a 15‐min passive head‐up tilt after exercise (Halliwill et al. 2014). However, it is unknown if post‐exercise reductions in cerebral perfusion lead to visual‐cognitive deficits during presyncope. Such a scenario would be of concern to populations such as emergency workers, or warfighters, who must perform complex tasks and make quick decisions in moments after vigorous physical activity resembling exercise, when vulnerability to reductions in cerebral perfusion and syncope are greatest (Luttrell and Halliwill 2015).

In general, post‐exercise syncope is “neurally mediated syncope” (also known as vasovagal or neurocardiogenic syncope), with hallmarks of paradoxical bradycardia and peripheral vasodilation, during recovery from exercise (Freeman et al. 2011). The primary trigger of neurally mediated syncope is unknown; however, a sustained reduction in cardiac preload seems a prerequisite. After exercise, several mechanisms act in parallel and result in reduced preload and cardiac output. Immediately after exercise there is augmented venous pooling in dependent regions of the body. Augmented venous pooling is the combined result of sustained arterial vasodilation of the previously active skeletal muscle, combined with a reduced skeletal muscle pump, and increased hydrostatic pressure gradients due to upright posture (Pollack and Wood 1949; Krediet et al. 2004; Halliwill et al. 2013, 2014). This transient reduction in cardiac output, if large enough in magnitude, might lead to decreased autoregulatory capacity in the cerebral circulation (Halliwill et al. 2014). Further, any impact of post‐exercise hypotension on cerebral perfusion may be exacerbated after high‐intensity exercise by the presence of a hyperventilation‐induced hypocapnic cerebral vasoconstriction (Rasmussen et al. 2006; Halliwill et al. 2014; Lacewell et al. 2014). Therefore, in the setting of upright post‐exercise recovery, we expect cerebral perfusion to be transiently decreased.

Neurally mediated syncope is usually preceded by symptoms of dizziness, light‐headedness, tunnel vision, and nausea (Freeman et al. 2011). These symptoms are thought to originate from a reduction in cerebral blood flow and oxygenation (Rossen et al. 1943; Van Lieshout et al. 2003). If a reduction in cerebral blood flow and oxygenation occur with neurally mediated syncope after exercise, it may also have detrimental effects on visual‐cognitive function during presyncope. On the other hand, a positive effect on visual‐cognitive performance has been demonstrated during an exercise bout, usually when subjects are in a seated position, which is generally attributed to increases in physical arousal (Brisswalter et al. 2002; Tomporowski 2002). However, changes in visual‐cognitive function during the exercise recovery period, when cardiovascular and cerebrovascular homeostasis are severely challenged, are not well defined. Therefore, the purpose of this experiment was to determine if reductions in cerebral blood velocity induced by maximal anaerobic exercise and head‐up tilt affect visual‐cognitive function prior to the onset of syncope. It was hypothesized that a modified Wingate test followed by a head‐up tilt would reduce cerebral blood velocity and result in visual‐cognitive deficits prior to the onset of syncope.

## Methods

This study was approved by the Institutional Review Board of the University of Oregon, and conformed to the principles of the Declaration of Helsinki. Each volunteer gave written and informed consent before participation. Prior to undergoing the experimental protocols, subjects completed a screening visit that was intended to familiarize them with all testing procedures, including the reaction time test of visual‐cognitive function.

### Subjects

Subject characteristics are summarized in Table 1. Twenty healthy, nonsmoking, normotensive subjects (10 men and 10 women) between the ages of 19 and 34 years participated in the study. Subjects were instructed to abstain from caffeine and alcohol for 12 h, and food for 2 h prior to the study. Based on the subjects’ self‐reported exercise habits over the previous month, they were classified as sedentary to recreationally active (Baecke et al. 1982; Kohl et al. 1988). Subjects completed a near‐fainting experiences index (Schrezemaier et al. 2005), to ensure they were not prone to syncope and had normal orthostatic tolerance. Due to the nature of the visual reaction time test, subjects were instructed to wear contact lenses to correct to normal vision if needed. Subjects were taking no over‐the‐counter or prescription medication other than oral contraceptives. Female subjects were studied during the early follicular phase of their menstrual cycle to minimize any potential cardiovascular effects of sex‐specific hormones and had a negative pregnancy test before completing the study.

**Table 1:** Subject characteristics

	Mean ± SD	Range
Age (years)	22.9 ± 3.6	19–34
Height (cm)	170.0 ± 8.8	159–190
Weight (kg)	64.0 ± 12.2	144.8–90.0
Body mass index (kg m^−2^)	21.9 ± 2.5	17.4–27.7
Baecke sport index (arbitrary units)	3.1 ± 0.6	2–4
Physical activity index (MET h week^−1^)	31.1 ± 11.2	8–50.1
Near‐fainting experiences index (arbitrary units from 0 to 20)	1.9 ± 1.8	0–5

MET, metabolic equivalents; *n* = 19.

### Experimental protocol

Studies took place in a thermoneutral environment (21.7 ± 1.1°C). At the start of the protocol, subjects sat upright and were instrumented for the measurement of arterial pressure, heart rate, end‐tidal CO_2_, and cerebral blood velocity. The subjects then stepped onto an electronic tilt table with footboard support (Colin Medical Instruments Corporation, Valley City, ND) that was tilted to 60° head‐up. Testing started within one minute of being positioned on the tilt table, a pre‐exercise measurement period of 8 tests (16 min) was completed where arterial pressure, heart rate, end‐tidal CO_2_, and cerebral blood velocity were measured continuously. Visual‐cognitive function was assessed in 2‐min test blocks during the head‐up tilt both before and after exercise. Upon completion of pre‐exercise measurements, subjects were seated on a cycle ergometer (Excalibur Sport V2; Lode BV, Groningen, The Netherlands) and performed a 5‐min warm‐up at a moderate resistance (100 W for males and 75 W for females) at a self‐selected pedaling cadence. Immediately following the warm up, subjects completed a modified (1‐min) Wingate test of anaerobic power. The torque factor was set to 0.63 Nm for male subjects and 0.60 Nm for female subjects (Wingate for Windows software version 1; Lode BV, Groningen, The Netherlands). Instrumentation was left in place throughout exercise to facilitate quick transitioning between exercise and post‐exercise measurements. Following the Wingate, subjects were immediately returned to the 60° head‐up position and re‐instrumented for visual‐cognitive function assessment and end‐tidal CO_2_. Post‐exercise measurements were started within one minute from the end of exercise, including continuous measurements of arterial pressure, heart rate, end‐tidal CO_2_, and cerebral blood velocity, which were again measured in the head‐up position until the subject was unable to continue or 16 min had passed.

Head‐up tilt tests were terminated if arterial pressure fell markedly (≥10 mm Hg) or heart rate slowed suddenly (≥10 beats) within one minute. In addition, subjects were asked to rate symptoms related to hypotension and cerebral hypoperfusion such as lightheadedness, nausea, and visual disturbances. Symptom scores were obtained every 2 min, each on a scale of 0–3 with 0 being no symptoms and 3 being the worst symptoms. Head‐up tilt tests were terminated if a subject reached either a 3 on any one symptom scale or a sum of 4 on the three combined scales.

### Measurements

#### Heart rate, arterial pressure, and systemic hemodynamics

Heart rate and arterial pressure were monitored throughout all experimental procedures. Heart rate was monitored using a 3‐lead electrocardiogram (Cardiocap/5 Critical Care Monitor; Datex‐Ohmeda, GE Healthcare, Helsinki, Finland). Arterial pressure was measured at the right brachial artery by automated auscultation (Tango+, SunTech Medical, Raleigh, NC) and mean arterial pressure was calculated, using the equation MAP = (SBP + 2 * DBP)/3. Finger photo‐plethysmography (Finometer; Finapres Medical Systems BV, Arnhem, the Netherlands) of the middle finger of the left hand, was used to monitor changes in blood pressure to determine termination of tilt test.

#### Cerebral blood velocity

A transcranial Doppler ultrasound (Spencer Technologies model ST3 with Marc 600 Headframe, Redmond, WA) was used to assess blood velocity in the middle cerebral artery. The ultrasound probe was secured with headgear at a constant angle to insonate the right middle cerebral artery over the temporal window and was adjusted for optimal signal intensity.

#### End‐tidal CO_2_

A capillary line connected to a nasal cannula was used to sample end‐tidal CO_2_ as a percentage (CardioCap/5 Critical Care Monitor, Datex‐Ohmeda, GE Healthcare, Helsinki, Finland) and corrected to partial pressure (mmHg) of CO_2_ using barometric pressure. To ensure accurate measurement, subjects were instructed to breathe primarily through their nostrils.

#### Reaction time and visual‐cognitive function

A visual reaction time test was used to assess visual‐cognitive function, similar to what has been used previously (Brisswalter et al. 2002; Tomporowski 2002). A custom concave metal frame positioned an array of computer‐controlled LED lights at the subject's eye level and at a distance of 60 cm from the subject's forehead. A single yellow light was located directly in front of the subject (midline), and to each side of the subject, pairs of red and green lights were located both centrally (10° from midline) and peripherally (50° from midline). During each visual‐cognitive function test block, which lasted 95 sec, subjects focused on the midline yellow LED which was constantly illuminated throughout the test. Subjects were instructed to respond, each time a green LED was illuminated, by releasing a micro‐switch with their right thumb as fast as possible, while disregarding illumination of red LEDs. The eight green and red LEDs were programmed to illuminate briefly (100 msec) in a balanced randomized order, with a balanced randomized duration (between 2500 and 3430 msec, in 30 msec increments) between each illumination. After each visual‐cognitive function test, blood pressure was measured using automated auscultation, and subjective symptom scores were obtained, such that a test block and these additional measures were repeated in 2‐min cycles. Thus, eight visual‐cognitive function test blocks were completed pre‐exercise during the 16‐min head‐up tilt. Up to eight visual‐cognitive function tests were completed post‐exercise, determined by the termination criteria described earlier.

### Data analysis

Continuous signals for electrocardiogram, capnogram, and transcranial Doppler velocity were sampled at 250 Hz, using a commercial analog‐to‐digital data acquisition system (Windaq, Dataq Instruments, Akron, OH).

#### Reaction time and visual‐cognitive function

For every 95 sec, visual‐cognitive function test block, reaction time, and error rates were calculated for responses to cues provided in the central and peripheral visual field. The reaction time, measured from the onset of an LED stimulus to the release of the thumb switch, was averaged for all correct responses to green LED stimulus. Error rate was calculated as the sum of incorrect, premature (<100 msec), late (>500 msec), and missed responses to the visual stimuli. Error rate is represented as a percent of total possible responses.

#### Statistics

In order to maximize statistical power, male and female subjects were analyzed as a single group. Hemodynamic and other outcome variables were analyzed with a two‐way repeated measures ANOVA (condition vs. time) with a priori contrasts of specific condition‐time combinations (SAS v9.2; SAS Institute, Inc., Cary, NC) and expressed as mean ± SE where group means are directly compared. The hypotheses were related to changes in cerebral blood velocity and visual‐cognitive function prior to the onset of syncope. These statistical tests were conducted using only data that preceded the development of syncope. In addition, the pre‐exercise values were averaged for each subject and compared to post‐exercise values at each time point by correlational analysis (Pearson Correlation Coefficient). Throughout all statistical testing, differences were considered significant when *P *<* *0.05.

## Results

**Figure 1:**
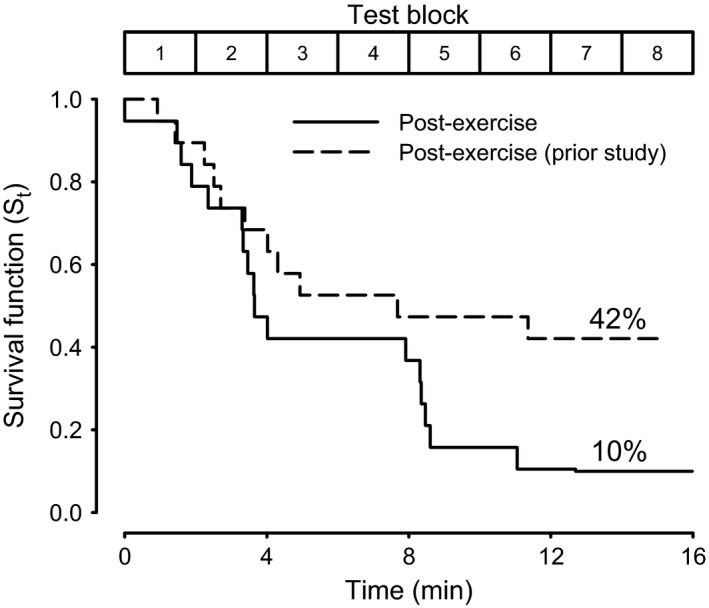
Survival function. Proportion of subjects completing each stage of the head‐up tilt shown as a survival function. Solid line, post‐exercise; dashed line, previous study using similar methodology (*n *=* *19; Reproduced from [Lacewell et al. 2014]).

Of the 20 subjects that participated in this study, 19 were able to complete the eight pre‐exercise test blocks, one subject could not withstand 16‐min head‐up tilt pre‐exercise, and was excluded from further analysis. Subjects tolerated post‐exercise head‐up tilt for variable durations; however, two of the subjects were able to complete the entire 16 min after exercise. Figure 1 demonstrates post‐exercise subject tolerance over time, illustrated as a survival function. Figure 1 also shows results from Lacewell et al. (2014), which used a similar exercise model but with a 2‐min supine rest prior to tilt. The general pattern of presyncope was similar, but subjects were more susceptible to presyncope without the supine rest preceding the head‐up tilt.

### Hemodynamic and physiological measurements

**Figure 2:**
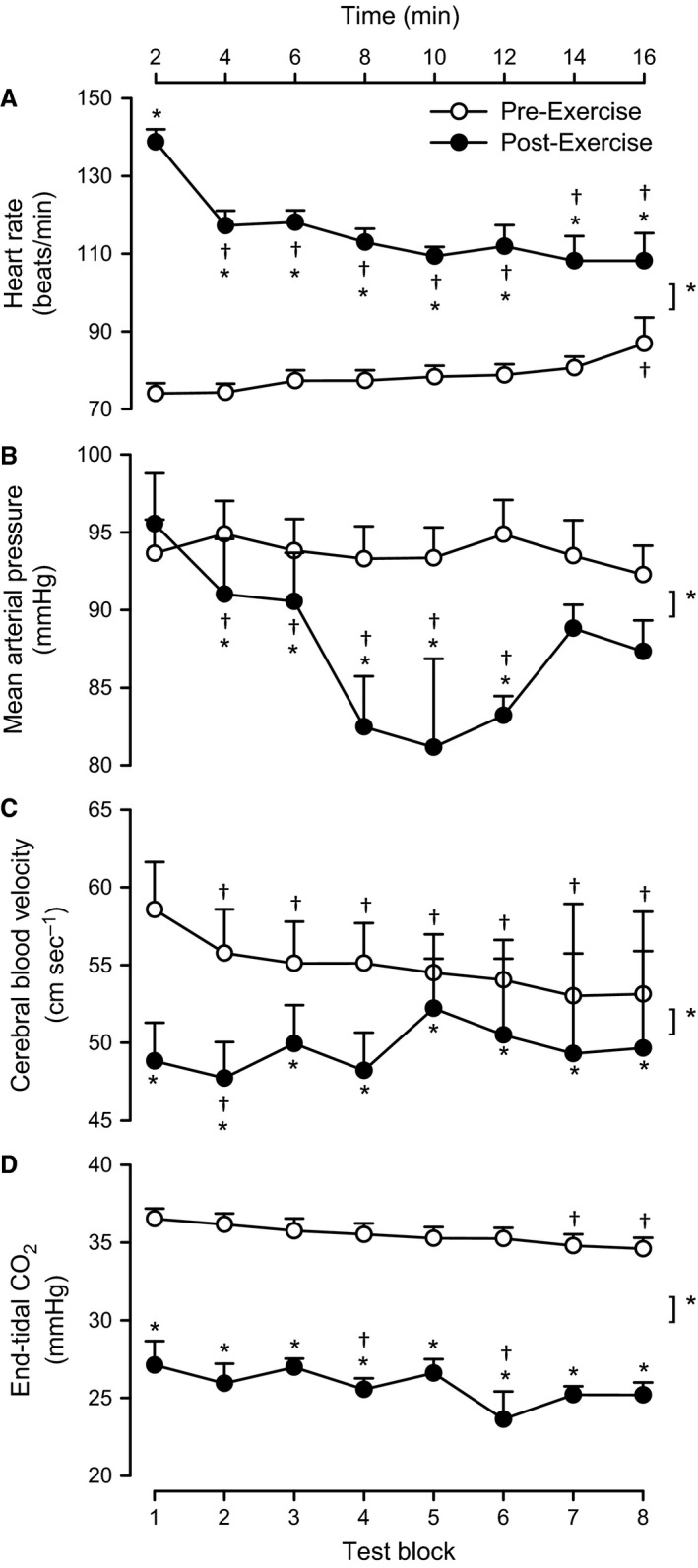
Hemodynamic and physiological variables across time during each of the two head‐up tilt tests (pre‐ and post‐exercise). Heart rate, mean arterial pressure, cerebral blood flow velocity, and end‐tidal CO_2_ averaged across each test block during a head‐up tilt performed before (open circles) and after exercise (closed circles). Values are means ± SE (*n* = 19). ^†^ *P < *0.05 versus the first test block in the same condition (either before or after exercise). *P < 0.05 before versus after exercise.

Figure 2 shows the key hemodynamic and physiological variables across time during each of the two head‐up tilt tests (pre‐ and post‐exercise). As shown in Figure 2A, prior to exercise, heart rate increased across the period from 74.0 ± 2.6 beats/min during the first test block to 86.9 ± 6.7 beats/min during the last test block (*P < *0.05). After exercise, the heart rate was increased compared to pre‐exercise (*P < *0.05), but subsequently decreased from the first test block (138.8 ± 3.2 beats/min) to the last test block (108.2 ± 7.1 beats/min; *P < *0.05).

As shown in Figure 2B, prior to exercise, mean arterial pressure did not differ from the first test block (93.6 ± 2.2 mmHg) to the last test block (92.3 ± 1.9 mmHg; *P = *0.49). After exercise, mean arterial pressure was similar to before exercise (*P = *0.34), but decreased from the first test block (95.5 ± 3.2 mmHg) to the sixth test block (83.2 ± 1.2 mmHg; *P < *0.05).

As shown in Figure 2C, prior to exercise, cerebral blood velocity decreased from the first test block (58.6 ± 3.1 cm sec^−1^) to the last test block (53.1 ± 2.8 cm sec^−1^; *P < *0.05). Cerebral blood velocity was significantly decreased post‐exercise when compared to pre‐exercise measures (*P < *0.05). During the post‐exercise period, cerebral blood velocity varied more compared to the pre‐exercise period due to increased subject drop‐out, but remained lower throughout the post‐exercise period by 12.7 ± 4.0% (*P *<* *0.05).

As shown in Figure 2D, prior to exercise, end‐tidal CO_2_ decreased across the period from the first test block (36.5 ± 0.7 mmHg) to the last test block (34.6 ± 0.7 mmHg; *P < *0.05). After exercise, end‐tidal CO_2_ was reduced compared to pre‐exercise (*P < *0.05), and did not differ from the first test block (27.1 ± 1.5 mmHg) to the last test block (25.2 ± 0.8 mmHg; *P = *0.15).

### Reaction time and visual‐cognitive function measurements

**Figure 3:**
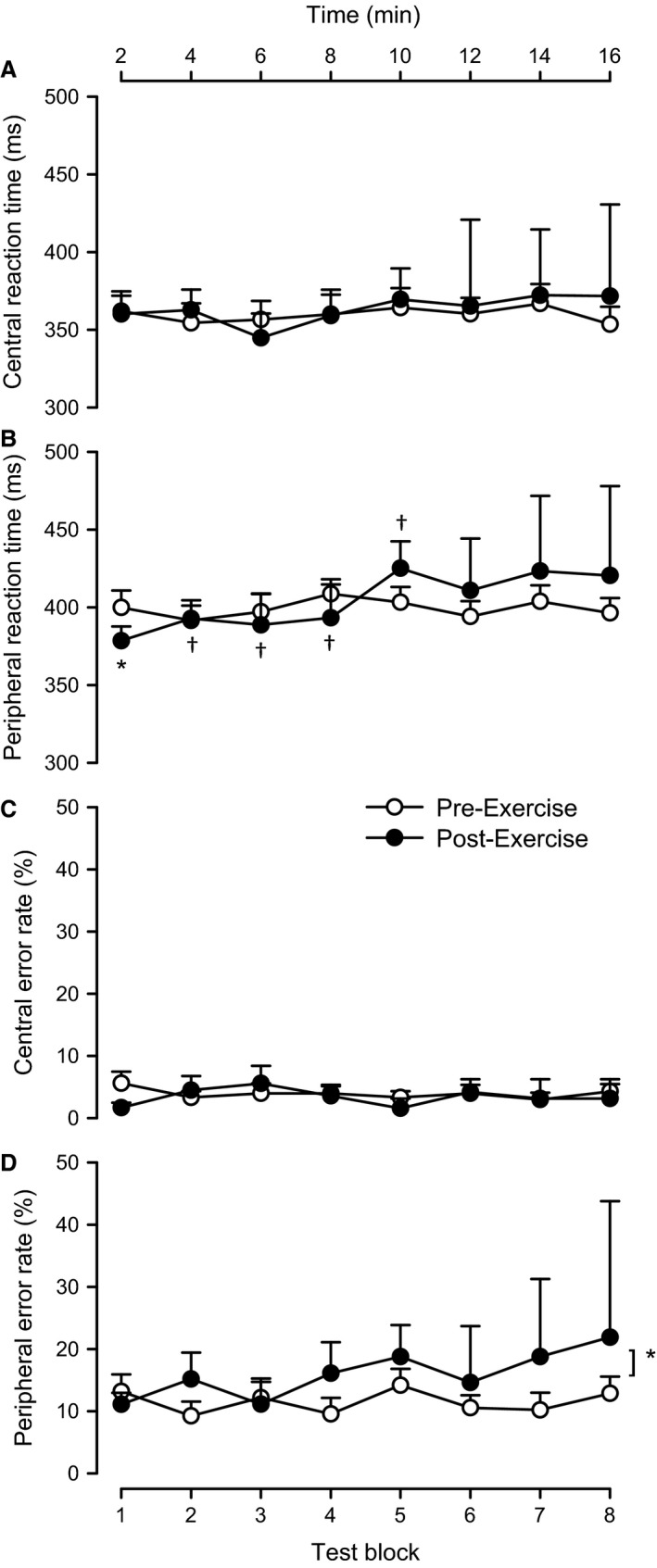
Reaction time and cognitive function measurements across time during each of the two head‐up tilt tests (pre‐ and post‐exercise). Central visual field reaction time, peripheral visual field reaction time, central visual field error rate, and peripheral visual field error rate averaged across each test block during a head‐up tilt performed before (open circles) and after exercise (closed circles). Values are means ± SE (*n* = 19). ^†^ *P < *0.05 versus the first test block in the same condition (either before or after exercise). *P < 0.05 before versus after exercise.

Figure 3 shows key visual‐cognitive function and reaction time variables across time during each of the two head‐up tilt tests (pre‐ and post‐exercise). As shown in Figure 3A, prior to exercise, the reaction time for responses to cues in the central visual fields did not differ from the first test block (362 ± 13 msec) to the last test block (354 ± 11 msec; *P = *0.19). After exercise, the reaction time in the central visual fields did not differ from pre‐exercise (*P = *0.35), and did not differ from the first test block (360 ± 12 msec) to the last test block (372 ± 59 msec; *P = *0.45).

As shown in Figure 3B, prior to exercise, the reaction time for responses to cues in the peripheral visual fields did not differ from the first test block (400 ± 11 msec) to the last test block (396 ± 10 msec; *P = *0.59). We note that reaction times were slower in the peripheral visual field than in the central visual fields by 11.6 ± 1.5% (*P *<* *0.05) when averaged across the entire testing period. After exercise, the reaction time in the peripheral visual field did not differ from pre‐exercise (*P = *0.48), and did not differ from the first test block (378 ± 9 msec) to the last test block (420 ± 58 msec; *P = *0.17).

As shown in Figure 3C, prior to exercise, error rates in the central visual fields did not differ from the first test block (5.6 ± 1.8%) to the last test block (4.3 ± 1.2%; *P = *0.41). After exercise, error rates in the central visual fields were unchanged (*P = *0.15), and did not differ from the first test block (1.6 ± 0.8%) to the last test block (3.1 ± 3.1%; *P = *0.86).

As shown in Figure 3D, prior to exercise, error rates in the peripheral visual fields did not differ from the first test block (13.2 ± 2.7%) to the last test block (12.8 ± 2.7%; *P = *0.89). We note that error rates were 3 times greater (3.0 ± 0.9; *P *<* *0.05) in the peripheral visual field than in the central visual fields when averaged across the entire testing period. After exercise, error rates in the peripheral visual fields were higher than pre‐exercise (by 63 ± 29%; *P < *0.05), and did not differ from the first test block (11.1 ± 1.9%) to the last test block (21.9 ± 21.9%; *P = *0.19).

Since subject drop‐out increased as time from exercise increased, several methods of analysis were employed to ensure an accurate portrayal of responses, with a focus on peripheral error rates. One such method was to compare averaged pre‐exercise data and to each subject's last post‐exercise measurement. Using this approach, we saw an increase in peripheral error rates from 10.8 ± 1.2% (average pre‐exercise) to 17.8 ± 3.8% (last post‐exercise test, *P *<* *0.05). We also note that, compared to averaged pre‐exercise values, a weak negative correlation was observed between cerebral blood velocity and error rate in the peripheral visual field (*r* = −0.25; *P *<* *0.05).

## Discussion

Passive standing, or head‐up tilt, is a physiological challenge which can lead to cardiovascular collapse in the form of pre‐syncopal signs and symptoms, or even frank syncope, if orthostasis is maintained (Blomqvist and Stone 1983). This physiological stress can be exaggerated following exercise, as there is an increased incidence of presyncope during head‐up tilt after exercise (Eichna et al. 1947; Lacewell et al. 2014), and it is estimated that presyncope will occur in 50–80% of healthy individuals if subjected to a 15‐min passive head‐up tilt after exercise (Halliwill et al. 2014). Signs and symptoms of presyncope have been attributed to reductions in cerebral perfusion and oxygenation of the brain (Rossen et al. 1943). However, it is unknown if post‐exercise reductions in cerebral perfusion associated with presyncope lead to visual‐cognitive deficits. This is of concern to populations such as emergency workers, or warfighters who must perform coordinated tasks and make quick decisions in moments after exercise when vulnerability to syncope is the greatest (Luttrell and Halliwill 2015). The results of the present study demonstrate that while the reaction time to a visual cue is well maintained in the face of orthostatic challenge after exercise, errors made while responding to visual cues in the peripheral visual field occur at higher rates (+63%) under these circumstances.

### Situations that affect visual‐cognitive performance

Acute exercise and its effect on visual‐cognitive performance has been extensively studied, but not in the presence of a superimposed orthostatic challenge. In contrast to the current study, which focused on post‐exercise effects, most other studies have focused on visual‐cognitive effects during exercise. These studies indicate a positive effect of acute exercise on cognition. The positive effect has been reported to be intensity dependent, represented by an “inverted U” model, where both low and high duration and intensity of exercise have less benefit on cognition compared to moderate exercise (Tomporowski 2002). The increase in cognitive function seen during acute exercise has been attributed to increased arousal (Tomporowski and Ellis 1986; Tomporowski 2002) Therefore, the effect of acute exercise on cognitive performance differs by intensity and duration of exercise as well as the type of cognitive task; and has been reviewed thoroughly (Tomporowski and Ellis 1986; Brisswalter et al. 2002; Tomporowski 2002; Chang et al. 2012).

The stressors of the current study differ from prior studies looking at exercise and cognitive function, and have more in common with the visual‐cognitive performance research on acute hypoxia, altitude, and pilots subjected to high‐G maneuvers, conditions which compromise global cerebral oxygen delivery. Acute hypoxia and high altitude (Vaernes et al. 1984; Kida and Imai 2015) are both associated with impaired visual‐cognitive performance due to reduction in arterial oxygen content. Studies examining visual‐cognitive function often differ in methodological approach which can make interpreting results difficult. Lie et al. found acute bouts of mild to moderate hypoxia negatively affect the visual reaction time, however error rate remained unchanged (Li et al. 2000). The present study found the reaction time was left unchanged while error rates increased. Taken together, we can conclude that visual‐cognitive function will be impaired when oxygen delivery to the brain is decreased, regardless of the mechanism. High‐G maneuvers and orthostatic challenge generate cerebral hypoxia via reductions in perfusion, rather than arterial oxygen content (such as the present study), but with a similar effect on visual‐cognitive function (Vaernes et al. 1984; Kida and Imai 2015).

In many situations, especially during hypoxic conditions, loss of vision has been shown to precede loss of consciousness (Whinnery and Forster 2015). Additionally, hypoxia has been suggested to cause a wide variety of visual decrements, including impaired color vision (Connolly et al. 2008), slowed visual processing (Fowler et al. 1993), and reduced peripheral vision (Whinnery and Forster 2015). The visual system is amongst the highest energy‐consuming systems in the brain, which is partly due to the retina being one of the highest oxygen‐consuming tissues in the body. Thus, the visual system, and especially the retina, is highly sensitive to even minor changes in tissue oxygenation (Wong‐Riley 2010). Hypoxia has been shown to increase reaction times and error rates while interactions with light intensity seem to play a role (Fowler et al. 1993). The present study was conducted in a room with no outdoor light and used a consistent lighting pattern across all studies to minimize any interaction with light intensity.

Unique to the Wingate syncope test, the reduction in cerebral blood velocity after exercise is most likely due to a combination of exercise‐induced hyperventilation and post‐exercise hypotension. In the present study, hyperventilation resulted in hypocapnia, which we have noted previously in this model of exercise and orthostatic challenge (Halliwill et al. 2013). Hyperventilatory‐induced hypocapnia has been shown to reduce cerebral blood velocity, likely by vasoconstricting cerebral blood vessels (Romero and Cooke 2007; Coverdale et al. 2014; Verbree et al. 2014); however, it may increase retinal blood flow by vasodilating retinal blood vessels (Brinchmann‐Hansen and Myhre 1990). The current finding of decreased blood flow velocity in the middle cerebral artery following exercise may not equate to reduced perfusion of all areas of the brain. A limitation of the transcranial Doppler technology is the inability to quantify changes in arterial diameter, and in theory, changes in diameter could offset some of the changes in velocity. That said, cerebral conduit arteries constrict in response to hypocapnia, and a decreased blood velocity with or without a cerebral conduit artery constriction would result in a reduced brain blood flow. While we did not study motor performance in the present study, it is worth noting that hypocapnia has been shown to impair motor performance (Gibson 1978), which could further exacerbate the impact of poor visual‐cognitive function during recovery from exercise. However, reaction times remained unchanged for stimuli to both visual fields, suggesting little or no motor impairment in this model.

### What is the nature of the cognitive deficit during presyncope?

In the present study, we found reaction times were unchanged for visual‐cognitive responses following intense exercise. As indicated above, the majority of studies indicate a positive effect of acute exercise on the reaction time during similar visual‐cognitive tests. One possibility is that the cerebral hypoperfusion, which exists in our model of orthostatic challenge following exercise, had no effect on the reaction time and central processing of information which are required to respond correctly to the test. However, we would suggest another possibility, which is that the positive effect of exercise, reported by others, is being circumvented by cerebral hypoperfusion in this model. If this is the case, then it is easy to anticipate that some variation in experimental conditions could tip the scale toward improved or impaired reaction times during the combination of intense exercise and orthostatic challenge.

Although we did not observe changes in error rates in response to the visual‐cognitive test when stimuli were presented in central visual fields, there was a notable increase in error rates for stimuli presented in peripheral visual fields. The selective nature of this deficit suggests that it is visual‐sensory in nature, and not secondary to an impaired cognitive decision‐making process. Along these lines, it may be related to the primary role of the rod photoreceptor in the peripheral visual field, and the sensitivity of rod photoreceptors to hypoxia. Mild hypoxia compromises threshold sensitivity during dark adaptation (Connolly and Hosking 2006), likely due to an impairment in the regeneration of rhodopsin. It has been suggested that rod photoreceptors may be functionally hypoxic when breathing normal air at the sea level (Connolly and Hosking 2006), and thus, on the shoulder of a steep dose–response curve when perfusion is challenged by orthostasis after intense exercise. This hypoxia‐derived impaired peripheral vision may be counteracted, to some extent, by hypocapnia, which enhances visual sensitivity and contrast discrimination (Connolly and Hosking 2006).

In summary, a reduction in arterial pressure (post‐exercise hypotension) paired with hyperventilation‐induced hypocapnia occurred after exercise, and lead to a reduction in cerebral blood velocity. The aforementioned reduction in cerebral blood velocity occurred concurrently with an increased peripheral visual field error rate indicating that individuals experiencing pre‐syncopal symptoms after exercise are also experiencing visual‐cognitive deficits, which are likely attributable to a loss of peripheral visual function rather than overall decrements in cognitive function. These results are important in identifying and managing situations in which visual‐cognitive deficits may occur, to attenuate possibly dangerous situations. Specifically, individuals can be expected to be less likely to respond, or respond correctly, to visual cues in the periphery of their vision, during recovery from intense exercise.

### Presyncope versus syncope

It is worth noting that our results are based on observations prior to the onset of syncope. After exercise, a clinically significant reduction in arterial pressure termed post‐exercise hypotension occurs (Kenny and Seals 1993), which can become symptomatic and result in syncope if left unchecked (Halliwill et al. 2013). One possible outcome is a rapid collapse due to neurocardiogenic syncope, a response whose trigger remains poorly understood, but may stem from vigorous contraction of an under‐filled left ventricle. It has been suggested that the hyperdynamic cardiac contraction of an under‐filled ventricle triggers a Bezold‐Jarisch‐like reflex which causes a paradoxical bradycardia via increased parasympathetic/vagal outflow to the heart and sympathoinhibition to peripheral blood vessels, resulting in vasodilation (Krediet et al. 2004; Halliwill et al. 2014). However, others have proposed mechanisms of neurocardiogenic syncope originating in the central nervous system rather than the ventricles (Hainsworth 2003).

Regardless of the specific trigger for neurocardiogenic syncope, there is a reduction in cerebral perfusion after the Wingate syncope test that was employed in the current study, which is due to the post‐exercise hypotension combined with extreme hypocapnia. The hypotension is compounded by augmented venous pooling, which is the combined result loss of the muscle pump and prolonged orthostasis (Pollack and Wood 1949; Krediet et al. 2004; Barrett‐O'Keefe et al. 2013; Halliwill et al. 2014). Of greater impact on post‐exercise hypotension is the marked vasodilation in the previously active skeletal muscle. When enough muscle mass is recruited for exercise, vasoconstriction of the inactive muscle groups is insufficient to offset the rise in vascular conductance in the previously active muscle, and the overall rise in systemic vascular conductance is greater than what can be supported by the heart when preload is also compromised. The exercise model used in this experiment, a modified Wingate test, activates large amounts of muscle mass that tips the proverbial scale to induce an increase in vascular conductance that could not be maintained during the passive stand test without experiencing presyncopal symptoms (Lacewell et al. 2014).

Thus, the majority of subjects were not able to continue with head‐up tilt, and one can postulate that visual‐cognitive impairments would be become marked (in response to both central and peripheral visual cues) had we allowed subjects to continue to the point of syncope.

### Perspectives

The present study demonstrated a reduction in the ability for individuals to accurately respond to visual stimuli in their peripheral visual field after an acute bout of high intensity short duration exercise. The attenuated visual/cognitive function can be partly attributed to reductions in brain blood velocity, which occurred when individuals could not actively recover from exercise. Athletes have reported experiencing symptoms such as tunnel vision and lightheadedness after finishing a race and being stuck in a crowded finishing chute, sometimes ending in syncope. Other populations, such as emergency workers or warfighters, can be expected to find themselves in similar situations in moments after vigorous physical activity resembling exercise. These populations must perform complex tasks and make quick decisions in situations when vulnerability to reductions in cerebral perfusion and syncope are the greatest. This situation can be dangerous and become life threatening if an emergency worker cannot clearly see if the scene is safe, or a warfighter has to identify a threat versus an ally. Future research should focus on simple physical countermeasure that can be performed to minimize the effects of post‐exercise recovery on visual/cognitive function by engaging the muscle pump and maintaining adequate cerebral perfusion.

## Conclusion

There is a reduction in cerebral blood velocity following a modified Wingate test and head‐up tilt. The reduction in cerebral blood velocity following maximal anaerobic exercise contributes to visual‐cognitive deficits as evidenced by the concurrent increase in peripheral visual field error rate during presyncope. Thus, individuals may be less likely to respond, or respond correctly, to visual cues in the periphery of their vision, during recovery from intense physical activity or exercise.

## Conflict of Interest

None declared.
